# Metabolomics for the masses: The future of metabolomics in a personalized world

**DOI:** 10.1016/j.nhtm.2017.06.001

**Published:** 2017-03

**Authors:** Drupad K. Trivedi, Katherine A. Hollywood, Royston Goodacre

**Affiliations:** Manchester Institute of Biotechnology and School of Chemistry, University of Manchester, 131 Princess Street, Manchester M1 7DN, UK

## Abstract

Current clinical practices focus on a small number of biochemical directly related to the pathophysiology with patients and thus only describe a very limited metabolome of a patient and fail to consider the interations of these small molecules. This lack of extended information may prevent clinicians from making the best possible therapeutic interventions in sufficient time to improve patient care. Various post-genomics ‘(’omic)’ approaches have been used for therapeutic interventions previously. Metabolomics now a well-established’omics approach, has been widely adopted as a novel approach for biomarker discovery and in tandem with genomics (especially SNPs and GWAS) has the potential for providing systemic understanding of the underlying causes of pathology. In this review, we discuss the relevance of metabolomics approaches in clinical sciences and its potential for biomarker discovery which may help guide clinical interventions. Although a powerful and potentially high throughput approach for biomarker discovery at the molecular level, true translation of metabolomics into clinics is an extremely slow process. Quicker adaptation of biomarkers discovered using metabolomics can be possible with novel portable and wearable technologies aided by clever data mining, as well as deep learning and artificial intelligence; we shall also discuss this with an eye to the future of precision medicine where metabolomics can be delivered to the masses.

## 1 Introduction

Central to this review is the role of metabolomics within the clinical sciences and so metabolomics as a discipline is first introduced, along with the role of clinically useful biomarkers (small molecules). Following this we discuss metabolomics approaches for personalised and precision medicine and the future role of delivering metabolomics to the masses.

Whilst there are many definitions of metabolomics we consider that metabolomics is a multidisciplinary science that seeks to define the entire complement of small molecular weight molecules termed metabolites within a biological matrix of interest. Metabolomics has been readily applied to a vast array of biological matrices of pre-clinical and clinical medicine relevance, with perhaps not surprisingly the most common being blood plasma and serum as well as urine. These are not the only samples accessible to the clinician and many studies have also focussed on extending these measurements towards intact tissues. This is particularly important for cancer diagnostics as measuring the pathology directly is likely to yield pathophysiological information about the disease (*i.e.* the cause) rather than measuring circulating metabolites (*i.e.* the likely downstream effect). In addition, studies have also shown that it is possible to generate information-rich metabolomes from human saliva, breath, cerebrospinal fluid (CSF), broncho alveolar lavage (BAL), sweat, faeces (as well as other locations in the gastro-intestinal tract), semen, and amniotic fluid. Finally, some research has also cultured primary cells for mammalian cell-based models, which may be particularly important for ADME-Tox (adsorption, distribution, metabolism and excretion-toxicology) studies.

The term metabolomics was first coined in the late 1990s [1] and had its 18th anniversary last year [2]. Metabolomics has increased in popularity and applicability ever since. Metabolomics can no longer be described as a novel concept within the clinical arena and it is now emergent. A simple search of Web of Science (on 7th Feb 2017) for (metabolom* OR metabonom*) AND (clinical OR medicine) within the research topic field returns over 3700 articles. Within the range of ‘omic approaches (*i.e.* transcriptome, proteome) the metabolome is perhaps the most closely linked to the phenotype of the subject and thus, can report on disease status as well as the effect and response to external stimuli (*e.g*. drug therapy, nutrition, exercise, *etc*).

## 2 The role of biomarkers in clinics

To treat disease GPs, clinicians and health workers require diagnostic indicators of disease, which can be used not only to diagnose said disease but also to assess the applicability of therapeutic interventions. These indicators are referred to as biomarkers and the NIH definition of a biomarker is [3]:

“A characteristic that is objectively measured and evaluated as an indicator of normal biological processes, pathogenic processes, or pharmacologic responses to a therapeutic intervention.”

**Fig. 1:**
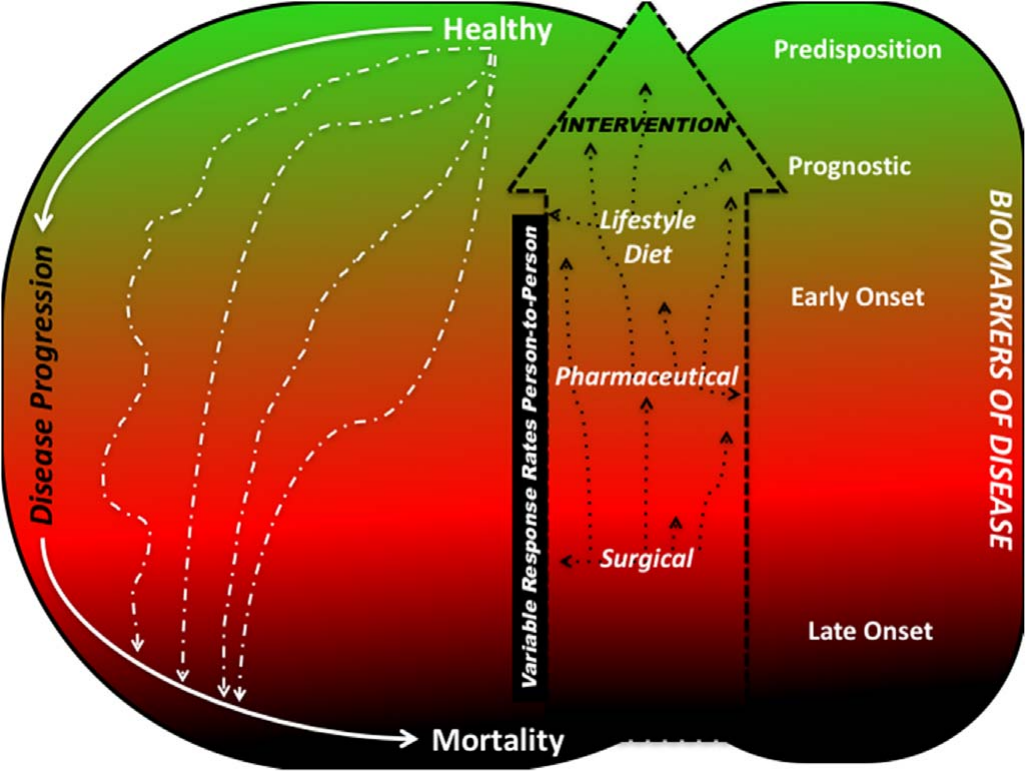
Figure illustrating disease progression (left hand side) along with the role of biomarkers on disease (right hand side) and how these may inform a range of personalised interventions.

The use of biomarkers to direct therapeutic intervention in terms of whether dietary and lifestyle interventions are necessary, or whether drugs are appropriate or if surgery is needed, is highlighted in Fig. 1.

A summary of clinically useful biomarkers used in clinical practice are provided in Table 1 (summarised from a test catalogue provided by Mayo Clinic, US available at http://www.mayomedicallaboratories.com). Whilst these biomarkers are very valuable for diagnosing disease there is an urgent need to have many more that are highly predictive and robust. There are many more biomarkers waiting to be discovered and this is a very active area of research in metabolomics.

**Table 1:** A selection of small molecule biomarkers and their clinical relevance; summarised from an available test list at the Mayo Clinic, US. The biological matrix investigated (U- urine, P-plasma, Sm-serum, Sa-saliva, Se-semen, WB-whole blood, BS-dried blood spot & C-CSF) and the analytical method/test applied is detailed.

## 3 Current positioning of metabolomics

With such popularity and regard, metabolomics as a discipline must look towards the future and begin to anticipate how the field can develop and transition into the modern-day world. As mentioned above metabolomics is now established, and whilst work is on-going in terms of technology and computational improvements, the method is now considered routine. Despite this maturity, the literature is unfortunately saturated with small-scale preliminary-type studies with many suffering from being poor in experimental design and thus any findings are likely to be false as they lack statistical robustness and validity [4]. This is not a unique feature of metabolomics and as nicely exemplified by George Poste [5] in his article entitled “Bring on the biomarkers”, whilst many biomarkers have been described in the academic literature comparatively few (well almost zero) have made it into the clinic. With reference to ‘omics and biomarker discovery one is often reminded of Henry Nix's famous statement [6]:

“Data does not equal information; information does not equal knowledge; and, most importantly of all, knowledge does not equal wisdom. We have oceans of data, rivers of information, small puddles of knowledge, and the odd drop of wisdom.”

**Fig. 2:**
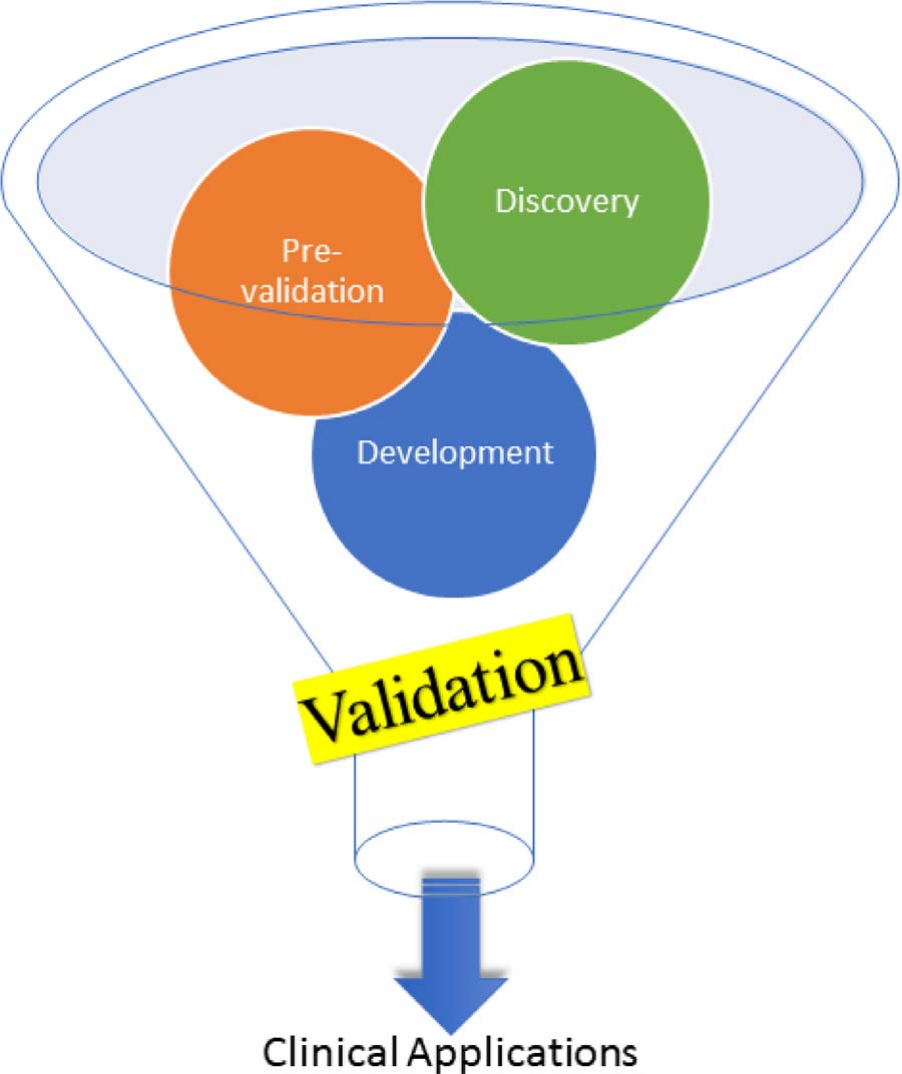
Schematic representation of the major steps for metabolomics biomarker discover. This initially starts out with a “Discovery” phase which involves in depth metabolomics assessment in (for example) case-control for disease stratification; this tends to be done on relatively small cohorts (*n* = 100 s). Following this a “Pre-validation” phase then repeats this untargeted metabolomics assessment in a different patient-control cohort (also of *n* = 100 s and preferably from a geographically distinct area from the first discovery phase). Following this there is an analytical “Development” phase for the assessment of the biomarker(s) discovered using lower cost technologies: this represents a shift from mass spectrometry or NMR spectroscopy to targeted chromatography or direct measurements using (for example) lateral flow devices. Finally using this faster and cheaper technology there is a “Validation” phase in large patient cohorts (*n* = 10,000/100,000 s) to assess the robustness of the biomarker(s) discovered.

With this in mind we believe that the field must drive forward towards the undertaking of large cohort multi-centre studies to enhance the discovery of biomarkers that have increased prospects of translation into point of care and rapid diagnostics; this biomarker discovery process is highlighted in Fig. 2, and of course is not limited to metabolites but any molecule.

Table 2 highlights several key metabolomics studies that have been aimed towards identifying biomarker candidates for an array of diseases. This table indicates the target disease of interest and the publication year, which illustrates the attempts made for biomarker discovery using metabolomics approaches, for a specific condition. It also summarises the number of control candidates and the number of diseased patients that were incorporated into the studies. Although these and other authors do not deliberately eschew obfuscation these numbers are often difficult to distinguish clearly within a manuscript. In addition, in some cases longitudinal studies are conducted whereby a patient is their own control. In order to have clarity in what was done within a study and what should be reported the Metabolomics Standards Initiative (MSI) initiated and subsequently published a series of papers on minimum reporting standards [7]. Within Table 2 the biomarker (or biomarker panels) that have been discovered within each study are documented and, we note if an independent validation has occurred within the same study which will of course increase confidence in the validity of said biomarker.

**Table 2:** Potential new metabolite biomarkers discovered and reported since 2000. Various sets of biomarkers have been proposed over the years for a number of diseases based on metabolomic investigations. Studies marked with an asterisk (*) indicates a further validation study that was included in the same publication.

**Disease/condition**	**Year of publication**	**Control subjects**	**Test subjects**	**Proposed biomarkers**
Abnormal savda	2008 [84]	20	110	Glycochenodeoxycholic acid and bilirubin
Acute coronary syndrome	2009 [90]	10	19	Citric acid, 4-hydroxyproline, aspartic acid, fructose, lactate, urea, glucose and valine
Acute kidney injury	2012 [132]	17	17	Dimethylarginine, pyroglutamate, lysoPC (selection of), acylcarnitine (selection of), phenylalanine, creatinine, homocysteine, methionine, arginine, tryptophan
Advanced liver fibrosis	2016 [165]	30	27	Panel inc: choline, glucose, glutamine, cysteine, histidine, citrate, acetoacetate
Alzheimer's disease	2010 [99]	20	20	Lysophosphocholine, tryptophan, phytosphingosine, dihydrosphingosine, hexadecosphinganine
Alzheimer's disease	2012 [127]	~52	~77	Desmosterol
Alzheimer's disease	2014 [148]	57	57	Arachidonic acid, *N, N*-dimethylglycine, thymine, glutamine, glutamic acid, and cytidine
Alzheimer's disease	2014 [151]	15	15	Alanine and taurine
Alzheimer's disease	2015 [164]	218	256	Sphinganine−1-phosphate, ornithine, phenlyllatic acid, inosine, 3-dehydrocarnitine, hypoxanthine
Asthma	2011 [110]	42	20	Panel inc: Adenosine, alanine, carnitine, formate, fumarate, glucose, histidine, taurine, threonine, succinate
Asthma	2013 [139]	26	39	methionine, glutamine, histidine
Atherosclerosis	2010 [103]	28	16	Palmitate, stearate and 1-monolinoleolglycerol
Autism*	2015 [161]	24	22	Methylguanidine, indoxyl sulfate, glucuronic acid, desaminotyrosine, guanidiosuccinate acid
Autism*	2016 [169]	63	73	Panel inc: decanoylcarnitine, pregnanetriol, uric acid, 9,10 epoxyoctadecanoic acid, docosahexanoic acid, docosapentanoic acid
Bladder cancer*	2011 [125]	16	28	Panel of 50+ differential metabolites
Bladder cancer	2014 [146]	121	138	Succinate, pyruvate, oxoglutarate, carnitine & acylcarnitines, phosphoenolpyruvate
Breast cancer	2010 [97]	50	50	Five unidentified biomarkers
Breast cancer	2012 [134]	34	80 (40 *vs* 40)	Palmitic acid, stearic acid, linoleic acid, FFA
Cardiovascular diseases	2014 [145]	/	67	Medium-and long-chain acylcarnitines, alanine
Chronic heart failure	2013 [143]	15	39	Lactate, creatine, glucose, glycoprotein, lipid species and amino acids
Chronic Hepatitis B	2006 [73]	50	37	Lysophosphatidyl choline and glycochenodeoxycholic acid
Chronic kidney disease	2011 [120]	13	18	Urinary neutrophil gelatinase-associated lipocalin
Chronic widespread musculoskeletal pain	2015 [160]	3736	1191	Epiandrosterone sulfate, dehydroisoandrosterone sulfate, androsterone sulfate, 3-(4-hydroxyphenyl) acetate, nonadecanoate
Colorectal cancer staging	2009 [87]	–	31	Panel inc: fatty acids, organic acids, sugars, steroid, fatty acid ester and pyrimidine nucleoside.
Colorectal cancer*	2010 [94]	110	112	Hydroxylated, polyunsaturated ultra-long-chain fatty acids
Colorectal cancer	2011 [117]	8	42	Free fatty acids and esterified fatty acids
Colorectal cancer	2016 [170]	254	320 (31)	Panel inc: octadecanoic acid, lactic acid, threonic acid, 3-hydroxy butanoic acid, serine, cysteine
Coronary artery disease	2012 [126]		2023	Dicarboxylacylcarnitines, medium-chain acylcarnitines, fatty acids
Coronary heart disease	2009 [88]	25	23	Saturated fatty acids, trans-fatty acid, n3 and n6 poly unsaturated fatty acids
Coronary heart disease*	2014 [41]	897	131	LysoPC (18:1), LysoPC (18:2), MG (18:2), SM (28:1)
Diabetes	2010 [106]	60	40	3-indoxyl sulfate, glycerophospholipids, free fatty acids and bile acids
Diabetic kidney disease	2012 [128]		52 (26 *vs* 26)	Acyl-carnitines, acyl-glycine and metabolites related to tryptophan metabolism
Diabetic mellitus and diabetic nephropathy	2011 [111]	30	120	Non-esterified fatty acids and esterified fatty acids
Diabetic nephropathy and type 2 diabetes	2009 [93]	25	41	Phytospingosine, glycine, lysine, dihydrosphingosine, leucine
Disorders of Propionate Metabolism*	2007 [78]	10	9	Propionyl carnitine, unsaturated acylcarnitine, γ-butyrobetaine, siovaleryl carnitine
Down syndrome	2015 [159]	93	23	Progesterone and dihydrouracil
Endometrial carcinoma	2016 [173]	25	25(10)	Porphobilinogen, acetlycysteine, *n*-acetylserine, urocanic acid, isobutylglycine
Gastric cancer	2016 [166]	40	83	Sucrose, dimethylamine, 1-methylnicotinamide, 2-furoylglycine, *N-*acetyl- serotonin, trans-aconitate, alanine, formate, and serotonin
Gastrointestinal cancer	2012 [129]	12	38	3-hydroxypropionic acid, pyruvic acid, L-alanine, glucuronolactone, L-glutamine
Healthy plasma metabolome	2008 [81]	269	–	300+ unique compounds
Hepatitis B*	2013 [140]	11	13	Tyrosinamide, biotin sulfone, hexanoic acid, 1-aminonaphthalene, 7-dehydroxycholesterol, azelaic acid
Hepatitis E and Hepatitis B	2011 [119]	18	32	Panel inc: L-proline, L-isoleucine, acetone, glycerol, glycine, biopterine, adenosine
Hepatocarcinoma	2011 [121]	38	41	1-methyladenosine
Hepatocellular carcinoma	2009 [92]	20	20	Panel of 18 metabolites inc: glycine, urea, threonine
High altitude pulmonary edema*	2015 [162]	35	35	Methionine, hypoxanthine, inosine, sphingosine, palmitoyl carnitine, C8 carnitine
Human hepatocellular carcinoma	2011 [116]	71	106	Bile acids, histidine, inosine, glycochenodeoxychoclic acid, glycocholic acid, taurocholic acid and chenodeoxycholic acid
Interstitial cystitis	2016 [172]	21	42	Oleic acid, 2-deoxytetronic acid, saccharic acid, phosphate, trehalose, erthronic acid, oxalic acid, sulfuric acid, cystine, lyxitol, lysine, histidine
Intestinal fistulas	2006 [76]	17	40	Glycochenodeoxycholic acid, glycodeoxycholic acid, taurochenodexycholic acid, taurodeoxycholic acid, lysophosphatidyl choline (C16: 0 and C18:2), phenylalanine, tryptophan and carnitine
IVF	2008 [85]	17	17	Glutamate and alanine/lactate ratios
Lepromatous leprosy	2011 [118]	10	13	Eicosapentaenoic acid, docosahexaenoic acid and arachidonic acid
Liver cirrhosis	2011 [113]	22	37	Lysophosphatidyl cholines, bile acids, hypoxanthine, stearamide, oleamide, myristamide
Liver failure due to Hepatitis B	2010 [104]	16	26	1-Lioleoylglycerophosphocholine or 1-linoleoylphosphatidylcholine
Lung cancer	2010 [108]	12	12	Lysophosphatidylcholines: lyso16:0, sn−2 lysoPC 16:0, sn−1 lysoPC 18:0, sn−1 lysoPC 18:1 and sn−1 lysoPC 18:2
Lung cancer	2011 [122]	29	33	A panel of 23 serum metabolites and 48 tissue specific metabolites
Lung cancer*	2014 [149]	536	469	Creatine riboside, cortisol sulfate, *N*-acetylneuraminic acid
Lung cancer*	2015 [157]	20	18	Maltose, ethanolamine, glycerol, palmitic acid, lactic acid,
Lung cancer	2015 [155]	55	41	Panel inc: trisaccharide phosphate, trihexose, nonanedioic acid, MG (22:2), tetrahexose
Lung cancer	2016 [167]	34	23 (11)	Isobutyl decanoate, putrescine, diethyl glutarate, cysteamine
Major depressive disorder	2012 [135]	25	26	Tryptophan, GABA and lysine
Major depressive disorder*	2015 [153]	59	60	Acyl carnitines, lipid metabolism and tryptophan
Malignant adrenal tumours	2011 [124]	45	102	Panel inc: metabolites from steroid metabolism pathways
Malignant Oligodendroglioma*	2008 [83]	10	24	Alanine, lipids, valine, the total choline compounds, proline, myoinositol, taurine, glutamine, glutamate, GABA, NAA, acetate, and creatine
Melamine-induced nephrolithiasis	2011 [123]	74	73	Proline, 5C-aglycone and hypoxanthine
Multiple sclerosis	2014 [150]	17	15	Choline, myo-inositol, threonate
Multiple sclerosis	2015 [156]	12	13	LPC (18:1), LPC (18:0), LPI (16:0), Glutamate
Muscle respiratory chain deficiencies	2015 [163]	13	24	AMP, *n*-acetyl asparagine, oxoglutaric acid, *n*-succinyl-L-L2.6 diaminopimelate
Nasopharyngeal carcinoma	2011 [115]	40	37	Kynurenine*, N*-acetylglucosaminylamine, *N*-acetylglucosamine and hydroxyphenylpyruvate
Oesophageal cancer	2013 [141]	26	89	Formate, acetate, short-chain fatty acids, GABA
Oesophageal squamous-cell carcinoma	2013 [144]	53	53	Phosphatidylserines, 12-oxo−20-dihydroxy-leukotriene B4, sphinganine 1-phosphate, LysoPC, phosphatidyl ethanolamine, phosphatidyl choline
Onchocerciasis*	2010 [105]	56	76	Panel of 14 inc: hexacosenoic acid, fatty acids, proteins, sterol lipids and phosphorylated sphingolipids
Oral cancer	2014 [152]	50	30	Phenylalanine & leucine
Oral, breast and pancreatic cancer	2010 [95]	87	128	betaine, choline, carnitine, glycerophosphocholine, cadaverine, putrescine, hypoxanthine, ethanolamine, trimethylamine and amino acids
Osteoarthritis*	2010 [98]	299	123	Valine to histidine ratio and leucine to histidine ratio
Ovarian cancer	2011 [112]	27	57	27-nor−5-beta-cholestane−3,7,12,24,25 pentol glucuronide
Ovarian cancer	2011 [114]	12	18	*N-*acetylasparate and *N-*acetyl-aspartyl-glutamate
Ovarian cancer*	2012 [131]	50	50	2-piperidinone, L-tryptophan, lysoPC (18:3), lysoPC (14:0)
Ovarian endometriosis	2012 [133]	52	40	Sphingomyelins and phosphatidylcholines
Paediatric acute liver failure	2009 [89]	20	20	α-NH2-butyric-acid (Aab) and Aab: leucine ratio
Pancreatic cancer	2016 [168]	40	40	Panel inc: palmitic acid, 1,2 dioeoyl GLP Na2, lanosterol, lignorceric acid, 1 oleoyl rac GL, chol epoxide, erucic acid
Parkinson's disease	2008 [79]	25	66	Uric acid and glutathione
Parkinson's disease	2009 [91]	37	43	Pyruvate
Parkinson's disease	2015 [158]	104	297	Cortisol, 11-deocycortisol, 21-deoxycortisol, histidine, urocanic acid, imadazoleacetic acid, hydroxyphenylacetic acid
Periodontal disease	2010 [101]	21	18	Inosine, lysine, putrescine and xanthine
Pre-eclampsia	2005 [72]	87	87	Three unidentified molecules
Pre-eclampsia	2017 [174]	20	20	Panel inc: PC (14:0/0:0), proline betaine, proline
Premature labour*	2010 [107]	16	39	Panel inc: Methyladenine, heptanedioic acid, *N-*acetylglutamine, glycerol, succinic acid, mannose
Prostate cancer	2010 [96]	30	40	Acylcarnitine and arachidonoyl amine
Prostate cancer	2013 [138]	178	331	Panel of 25 metabolites inc top 5: histidine, glycine, alanine, kynurenine, glutamate & glycerol−3-phosphate
Psoriasis	2017 [175]	15	14	Asparagine, aspartic acid, isoleucine, phenylalanine, ornithine, proline, lactic acid & urea
Rectal cancer	2013 [142]	43	127	Lactate, threonine, acetate, glutathione, uracil, succinate, serine, formate, lysine and tyrosine
Renal cell carcinoma	2010 [100]	13	32	Panel inc: acetate, glutamate, glutamine, glucose, tyrosine, histidine, phenylalanine, formic acid, alanine, lactate
Rheumatoid arthritis	2010 [102]	51	47	Cholesterol, lactate, acetylated glycoprotein and lipids
Rheumatoid arthritis	2011 [109]	20	25	Panel inc: Glyceric acid, hypoxanthine, histidine, threonic acid, methionine, cholesterol, threonine
Rheumatoid arthritis	2016 [171]	19	46	Arginine, aspartic acid, glutamic acid, phenylalanine, serine, threonine, methlynicotinamide
Schizophrenia	2006 [74]	70	82	Citrate, glutamine, acetate, lactate
Schizophrenia	2007 [77]	–	50	50 lipids including triacylglycerols, free fatty acids, phosphatidylethanolamine.
Schizophrenia*	2013 [137]	62	62	Glycerate, eicosenoic acid, beta-hydroxybutyrate, pyruvate, cysteine
Systemic inflammatory response syndrome (SIRS) & Sepsis	2012 [130]		143 (74 *vs* 69)	Acylcarnitines and glycerophosphatidylcholines (C10:1 and PCaaC32:0)
Type 2 diabetes	2006 [75]	45	78	Non-esterified and esterified fatty acids in plasma
Type 2 diabetes	2008 [80]	28	23	3-hydroxyhippuric acid
Type 2 diabetes	2008 [86]	time course study	75	Citrate, IL−8 and methyl-histidine and branched amino acid degradation products
Type 2 diabetes (T2DM) and Type 2 diabetic coronary heart diseases (T2DM-CHD)	2008 [82]	45	71 and 37 for T2DM & T2DM-CHD	Free fatty acid (C16:0, C18:1 *n*−9 and C18:2 *n*−6)
Type 2 diabetes & impaired fasting glucose	2013 [136]	1897	115 & 192 respectively	Panel inc: amino acids, lipids, carbohydrates (T2D) & panel of lipids, carbohydrates, amino acid plus urate & erythritol (IFG)
Type 2 diabetes mellitus	2015 [154]	300	300	Lipids, hexose sugars, purine nucleotide
Ulcerative colitis (UC) & Crohn's disease (CD)	2014 [147]	17	24 UC & 19 CD	Panel inc: N-acetylated glycoprotein, lactate, methanol, mannose, formate

It is clear from inspecting this Table 2 that there is a broad difference in the number of subjects included in these studies. The community is yet to decide what this number should be, but it should be noted and acknowledged that the availability of patients will greatly vary from disease to disease and equally access to valuable (sometimes very rare) samples will be limited. In this century alone, there have been more than 1600 publications (using a combined search of the above PLUS biomarker* from 2000 to date) that ‘claim’ to have discovered a biomarker using a metabolomics approach, which is nearly half of all papers surveyed! Although there are some exceptions, most of this research fails to acquire enough statistical power due to a limited sample size (<100 subjects in total) and almost none repeat the analysis in a further cohort and thus fail to demonstrate a lack of biomarker utility. We believe that these thwart the potential translation of metabolomics research into clinics. For instance, there is minimal-known translation of metabolomics biomarker discovery into clinics for the top five causes of death in the UK (Table 3) which include: ischaemic heart diseases, dementia and Alzheimer's disease, malignant neoplasms of trachea, bronchus and lung, chronic lower respiratory diseases and cerebrovascular diseases [8]. Malignant neoplasms, respiratory disease and ischaemic heart diseases are also three of the top five leading causes of death across Europe [9].

**Table 3:** Top 5 leading causes of death in men and women in England and Wales (2014).

**Men**	
Ischaemic heart diseases	36,293
Dementia and Alzheimer's disease	15,973
Malignant neoplasm of trachea, bronchus and lung	14,359
Chronic lower respiratory diseases	13,952
Cerebrovascular diseases	12,584
**Women**	
Dementia and Alzheimer's disease	33,153
Ischaemic heart diseases	24,057
Cerebrovascular diseases	19,127
Chronic lower respiratory diseases	14,181
Malignant neoplasm of trachea, bronchus and lung	11,309

Despite the above disease being of obvious importance we note the rapid rise of microorganisms as contributing to world-wide mortality. The obvious ‘culprits’ here being *Mycobacterium tuberculosis* and HIV, but with the almost meteoric rise in antimicrobial resistance (AMR) many normally harmless opportunistic pathogens will become increasingly important. Indeed it is predicted by 2050 that bacterial infections will kill more humans than cancer and heart disease [10]. Whilst it is accepted that there are many microbial interactions with the host cell microbiome and that man is a true superorganism [11] it is also notable that many common human disease may indeed have a microbial origin [12]. Metabolomics is likely to play a valuable role in understanding AMR and the host-pathogen interaction.

This review seeks to provide an overview of metabolomics in respect to diagnostic applications and demographic screening and present a futuristic perspective on the implementation of the field with novel portable and wearable technologies.

## 4 Is the future of healthcare simply personalized medicine?

**Fig. 3:**
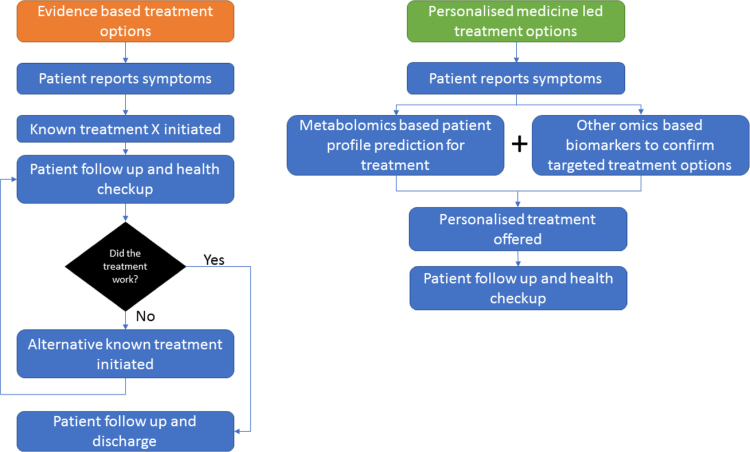
Flow diagram illustrating personalised medicine and highlighting the differences between Evidence-based *versus* Precision medicine-based approaches to disease treatment. As is clear the evidence-based approach is imprecise as it relies on the patient reporting progress to therapy. By contrast, precision medicine necessitates analytical measurements on the patient – typically from genetics (*viz*. SNPs) and metabolomics–and then using these to direct therapy.

Although personalised medicine is a generic entity relatively new to the field of healthcare research, it has of course been practiced for decades within a so-called evidence-based framework (Fig. 3). In evidence-based medicine an individual is treated for disease largely based on the most popular medicine. After the drug is taken for some time an assessment is made, with the desire to evaluate whether this has relieved symptoms (this may involve the measurement of a clinically useful biomarker (Table 1)). Based on this deterministic assessment the patient may then stay on the same drug, be diagnosed an alternate medicine, or be given a treatment to relieve side effects of the first drug. This process is slow and potentially dangerous to the patient. A much more desirable approach is to use precision medicine and this was brought to the forefront of attention when, during his 2015 State of the Union address President Obama announced that he was launching the Precision Medicine Initiative. This was heralded as a bold new research direction [13] with changing for biobank made available by NIH to support the initiative [14].

Precision medicine involves assessing the genotype (*e.g.* SNPs) and phenotype (*e.g.* metabolome) of the patient before they undergo any treatment (Fig. 3) and therefore relies on accurate analytical methods for directing therapy [15]. Biomarkers are needed that can accurately identify the underlying pathology as these may help understand the disease aetiology and thereby result in a precise treatment [16], [17]. Clearly the lack of suitable biomarkers currently holds back the wider implementation of personalised medicine [18]. This is where metabolomics plays a key role as an approach to discover a biomarker, trial its detection within a large diverse population and then translate its detection into cheaper, quicker and reliable methods that could be used by a wider audience [19]. As indicated above the main use of metabolomics as a tool is for biomarker discovery [20], [21], [22], [23], [24]. The closest representation of a disease phenotype is a key-driving factor for the increased use of metabolomics for biomarker discovery to understand disease pathologies and finding methods of cure, and as many diseases result in changes in human metabolism it makes sense to use a method that measures metabolism directly!

However, the focus of biomarker discovery should not only be for pathological cures but also for preventive screening of healthy individuals (Fig. 1), as earlier biomarkers may be useful in directing dietary and lifestyle changes prior to more radical surgical treatment. Within biomarker discovery this raises the tantalising idea that all healthy individuals should undergo some biomarker screen well before any disease is found so that any change in a biomarker(s) level is personalised; for example, someone with an already raised PSA level may not have prostate cancer and this higher PSA levels maybe indicative of an enlarged prostate as one ages [25]. Offering a well-designed screening program at a reasonable cost may not always be possible due to the numerous associated challenges; these include monetary limitations (labour and consumable costs) as well as ethical, legal and social considerations for an opt-in test. The risk-benefit ratio needs to be clearly defined per disease for a successful personalised screening [26]. Encouraging biomarker discoveries from within the plasma metabolome [27], serum metabolome [28], [29], [30], [31], urinary metabolome [32], [33], [34], [35] as well as the volatilome [36], [37], [38] show immense potential of dramatically reducing health risks in fatal health conditions such as cancer, congenital disease, heart diseases and respiratory diseases (Table 3). It should be noted that a key hurdle for translation of these biomarkers into a routine clinical test is the failure to **validate**. In the absence of a universally accepted procedure for metabolic profiling used for biomarker discovery, different sites use their own optimized procedures. Additionally, even if identical analytical platforms and routines are used, the inherent inter-laboratory variation will play a great role in detracting from the validity of a potential biomarker and there can never be certainty that the entire metabolome has been profiled with that said platform, and in fact it is accepted in the metabolomics community that there is no magic tricorder that measures everything [39]. Thus, there is always a **potentially** ‘better’ biomarker waiting to be discovered.

Some notable large scale and/or multicentre metabolomics studies have been successfully conducted (Table 2) to map the human serum metabolome [40], to identify biomarkers for incident coronary heart disease [41], and to study the response of *Aspergillus nidulans* to epigenetic perturbation with a hope to expedite the search for new pharmaceutical leads [42]. A correct balance needs to be considered between large scale *vs.* small sub-population focused studies where the risk is minimal but with maximum benefits [43], [44]. Due to the higher cost and effort involved in the analysis of samples by a standard metabolomics workflow, it is often tempting (albeit one could say lazy) to use a smaller sample size for biomarker discovery and pre-validation [45]. However, such studies which lack the required statistical power for confident biomarker assessment will entice anyone to start designing specific assays for assessments in large cohorts (Fig. 2).

Like all ‘omics which are data rich, metabolomics on humans is influenced by many confounding factors such as age, gender, ethnicity, diet *etc*. [40] and thus, large validation studies with suitable control cohorts must be used to remove any potential bias [4], [46]. Certain metabolites that alter with normal physiological changes may also be significantly different in a metabolomics study. By way of an example, citrate has been shown to increase with age [40] even in healthy individuals. A recent metabolomics study indicated amongst other metabolites that citrate was a significantly important biomarker for cancer [47]. However, since an increase in citrate could also be attributed to difference in mean age (17 cancer patients = 70 and 21 healthy controls = 60) rather than altered TCA cycle in cancer, in the absence of closely age matched case-control cohort such results need to be taken with caution before inferring pathological importance of such a biomarker.

Whilst the current perception is that screening large control groups of healthy individuals at the same time as diseased populations is not an option for validation studies, this position must change. Indeed, many people already use wearable technology for the assessment of their exercise levels, heart rate, blood oxygen levels, as well as sleeping patterns, so collecting data on ‘healthy’ individuals is not that maverick.

## 5 Metabolomics for the masses

With recent technological advancements in the form of affordable hardware (*e.g.* pedometers which include heart rate monitoring), health apps on smartphones, fitness bands and smart-watches, it is feasible to generate large amounts of useful health-related data even in healthy populations [48], [49]. These measurements are readily available on a personalised level and could be used to complement clinical studies. For example, in treatment regimens which may include nutritional and exercise advice.

The tantalising question is whether metabolomics could be delivered to the masses on a personalised level? Whilst mass spectrometry linked to chromatography is a very power metabolomics platform for biomarker discovery, it is laborious and expensive and therefore unlikely to be suitable for large-scale screening of very large populations (*i.e.* when *n* > 10,000, which is of course still small when we consider that the earth's population is estimated to be >7.5×10^9^; http://www.worldometers.info/world-population/). Of course, once a series of biomarkers are discovered and validated the scenario is different where one now knows the measurands and these can be detected and quantified using analytical chemistry. These can include methods based on:

• Lateral flow devices – much like the pregnancy test which is based on antibody detection of the appropriate antigen (*viz*., human chorionic gonadotropin (hCG));
• Dipstick approaches – for example the detection of nitrite for confirming urinary tract infections;
• Breath measurements for volatiles – for example ethanol detection and quantification using fuel cells for road side testing;
• Electrochemical detection – under skin glucose test is based on this and allows constant assessment of blood glucose that can be linked automatically to insulin injections [50].

**Fig. 4:**
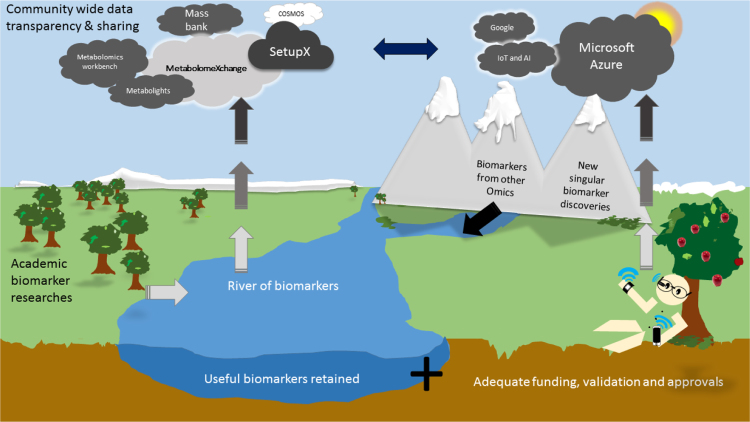
The future cycle of metabolomics precision medicine-based research and healthcare where academia, industrial partners, corporate data analytics work with patients’ wearable data collection devices to provide health monitoring solutions.

With the above in mind emerging technologies in metabolomics provide new platforms for high-throughput, highly sensitive, functional assays, biomarker discovery and offer opportunities for personalised medicine, complementing existing and emerging genomic, proteomic and transcriptomic technologies (Fig. 4). However, personalised medicine in the future could be better served when these biomarkers provide enough knowledge to translate them successfully into one or more types of wearable technologies that are readily available to an end user (as also illustrated in Fig. 4). Biosensors used in wearable technologies like smartphones [51], [52], smart-watches [53] for monitoring heart conditions, health bands, necklaces, glucose monitoring contact lenses [54], [55], headbands *etc.,* are excellent innovations transferring biomarker discovery onto a more individual level. Technological advances translating biochemical changes into physical signals is not something new, but in this age of bionics and biohacking [56], it is putting the technology in the hands of end user and thus able to boost the personalised medicine movement [57]. Although, we recognise that these devices should always be with continued consultation with a clinical practitioner who can advise the wearer; much like home testing for blood pressure is currently practiced.

Metabolomics studies seldom lack information about the studied bio-system, and although this science is described as data rich, as many metabolites are measured, it often lacks a sizable population. Being able to translate a subset of metabolite measurements onto a set of devices that not only validates the results but also provides other useful complementary information about the patient we believe is the next step forward.

Chemometric-based analyses following any metabolomics study can immensely benefit from such wealth of continual metabolite data and metadata obtained from a target cohort. When metabolomics for the masses does occur the data processing may need to depend on large computing power and data storage space and these could be stored securely and privately within an advanced cloud-computing environment [58]. In such a scenario, these measurements from different populations could be linked within the internet (so called internet of things (IoT)) allowing ensemble computation and for example epidemiological assessment of disease progression and spread (Fig. 4). This is not novel as the added benefit of current wearable biosensors is that the collected data are directly synced onto intelligent cloud services like Microsoft Azure or Google Cloud [59]. An individuals (health) data collected over time *via* portable (a set of wearables) devices has the potential of producing copious amounts of telemetry data that can be computed over the cloud, producing predictions for *future* health risks. For example the mPower, mobile Parkinson's Disease (PD) study that attempts to research the occurrence, presentation and management of PD symptoms *via* survey telemetry data using a smartphone app [60]. Another nice example is a smart-phone based application to monitor the association between pain and the weather for people suffering from rheumatoid arthritis [61]. Use of artificial intelligence (AI) for screening, decision making and management whilest not new, can be adapted for risk stratification, prevention and choice of treatment in the healthcare systems [62]. Recently, US Food and Drug Administration (FDA) has approved an AI based machine learning application for the use in clinics for making informed decisions about the health of heart where AI provides accurate measurements of the volume of each ventricle to physicians. This is thought to speed up and improve the decision making for heart surgeries [63]. There is an enormous potential for a happy marriage between metabolomics and AI or machine learning technologies that are driven by data. This is where metabolomics should aim to take personalised medicine to - not only being able to predict a persons current or near future health or globally screen for potential biomarkers - but to link that information to dynamic metadata from patients to predict further risks and disease prognosis (Fig. 1). This approach as opposed to evidence-based medicine (Fig. 3) will enable better health care outcomes instead of trial and error treatment regimes.

**Fig. 5:**
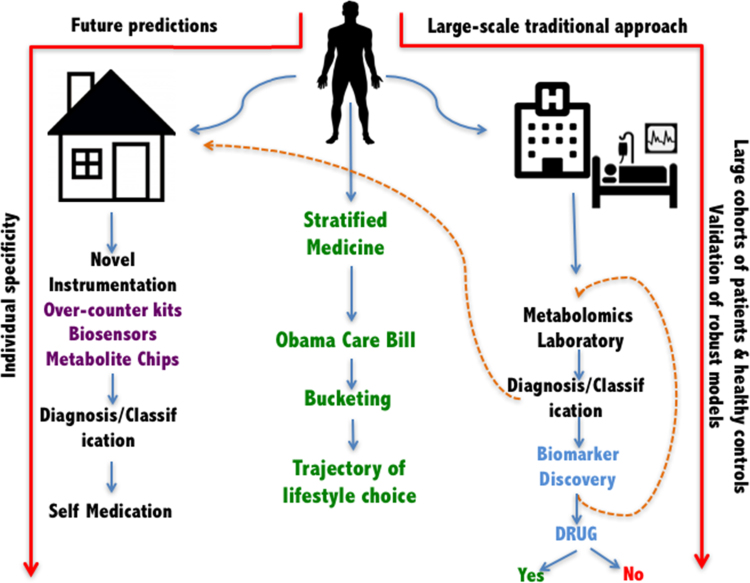
A potential future where the patient is at the centre of their own health care. Where research/omics data and clinical data (right sides) are combined with novel future wearable and at home testing to generate more precise and thus precision medicine-based diagnostics. Thus, bucketing patients with similar health profiles would aid clinics to differentiate those that need urgent medical intervention from those that will benefit more from change in lifestyle choices and non-medical aid. This approach can thus help identify subgroup(s) of patients with similar drug responses or disease profiles, enabling affordable care as proposed by the Obama Care Bill without excluding those with pre-existing health conditions (that are not deemed life threatening but manageable) or comorbidities.

A potential future scenario illustrating precision medicine were together the patient and physician are at the centre of the diagnostics is shown in Fig. 5, once the hurdles of costs, barriers to patient inclusion and ease of use are overcome [64]. On the right-hand side of this figure is the expected laboratory-based scenario where metabolomics data are a standalone set of information which may be frequently linked to other ‘omics data. These measurements are detailed and thus slow and usually reserved for the initial diagnostics often when disease is already apparent. This provides useful but limited retrospective information about a population. By contrast the left-hand side illustrates the role of self-testing at home which can occur much more frequently, and for some wearable devices constantly and in real-time. For example, using dipstick tests for diabetes may be a quicker assessment of glucose levels but as is already known by individuals with Type I diabetes lacks real-time prolonged monitoring of patient health. As mentioned above implantable devices are now available for real-time glucose sensing and when combined with a ‘health band’ which reports information on a patients sleep patterns, heart rate, and physical exercise schedules may lead to better management of the disease.

## 6 Conclusions

The future of metabolomics does not stop at personalised medicine itself. For the application of metabolomics in preventive medicine as well as screening, the world is your oyster. Indeed, metabolomics could play not only a crucial role in monitoring life on the Earth but also beyond [65]. NASA's recent famous twin study which was concluded last year will hopefully show a glimpse of how powerful and useful understanding the human metabolome can be [66], [67].

At present metabolomics is very much research laboratory-based and needs to move out of academic laboratories and into the clinic. As a step towards this the UK has established two Phenome centres [68], one in London and the other in Birmingham; time will tell whether these are successful but a real opportunity is presented for the large-scale use of metabolomics for preventive health care, disease diagnosis, disease monitoring as well as finding novel therapeutics on a personalised level, which will account for differences within each individual.

A recently published white paper demonstrates the strengths of metabolomics in shaping precision medicine [69], and we would urge all readers to dip into the text along with the accompanying Topical Issue published in *Metabolomics* on “Recent advances in Pharmacometabolomics: enabling tools for precision medicine” [70].

As the ancient proverb says:

“*Vita brevis, ars longa, occasio praeceps, experimentum periculosum, iudicium difficile*” [71]

which translates to:

“Life is short, and art long, opportunity fleeting, experimentations perilous, and judgement difficult.”

Thus, there is an urgent and somewhat imminent need for precision medicine! This will require appropriate infrastructure for *metabolomics for (and indeed on) the masses* and will require alterations in healthcare practices across the globe. Once delivered this may improve medicine, put the patient at the centre of the analysis, and allow for healthier lifestyles and efficient medication for each and every one of us.
